# Jaccoud's Arthropathy in Gout: An Unusual Association

**DOI:** 10.1155/2014/873189

**Published:** 2014-08-14

**Authors:** Tayana Donato Alves, Isabela S. Oliveira, Luiza C. Fadul, Mittermayer B. Santiago

**Affiliations:** ^1^Universidade Federal da Bahia, Rua Augusto Viana s/n, Canela, 40110-060 Salvador, BA, Brazil; ^2^Escola Bahiana de Medicina e Saúde Pública (EBMSP), Avenida Dom João VI 275, Brotas, 40290-000 Salvador, BA, Brazil; ^3^Serviços Especializados em Reumatologia da Bahia, Rua Conde Filho 117, Graça, 40150-150 Salvador, BA, Brazil

## Abstract

Jaccoud's arthropathy (JA) is a deforming arthropathy observed mainly in patients with systemic lupus erythematosus. We report the case of a 41-year-old Brazilian man with a 10-year history of intermittent monoarthritis followed by bouts of polyarthritis affecting large and small joints. He presented deformities typical of JA and subcutaneous nodules. Histopathological findings of a nodule biopsy were consistent with gouty tophi. To our knowledge, this is the first report of JA secondary to chronic gout.

## 1. Introduction

Jaccoud's arthropathy (JA), a deforming arthropathy, was initially described in patients with rheumatic fever (RF). Presently, it has been reported mainly in patients with systemic lupus erythematosus [1]. However, several case reports have associated this complication with other clinical conditions, including Sjögren's syndrome [2], dermatomyositis [3], scleroderma [4], sarcoidosis [5], and HIV infection [6]. Moreover, senescence JA is a variation described in normal elderly individuals [7].

Our aim was to present the first case of polyarticular gout with reversible hand deformities typical of JA and discuss its differential diagnosis.

## 2. Case Report

**Figure 1:**
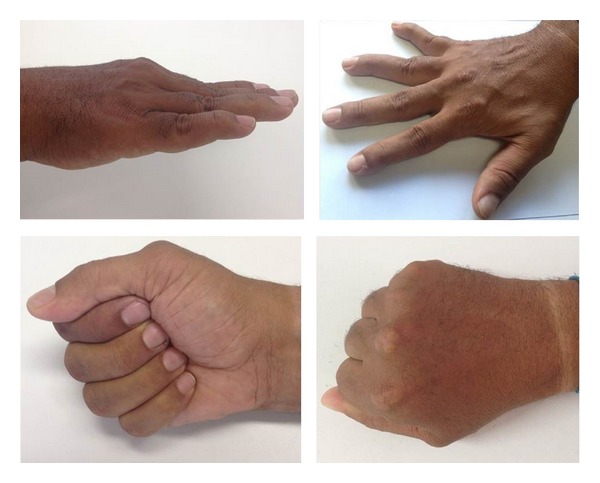
“Reversible”* swan-neck* deformities of the right hand.

**Figure 2:**
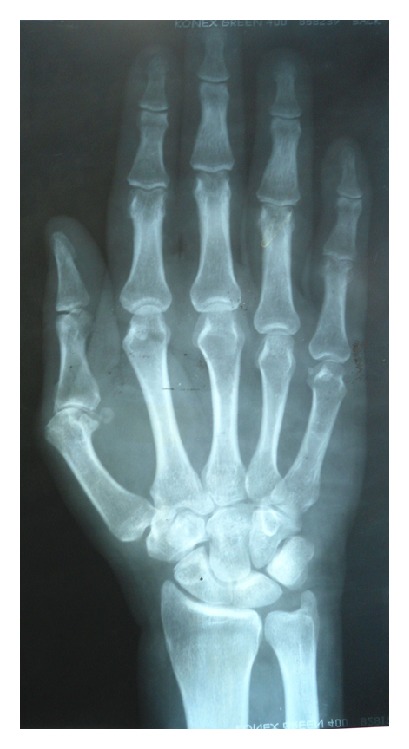
Plain radiograph of the right hand showing no bone erosions.

**Figure 3:**
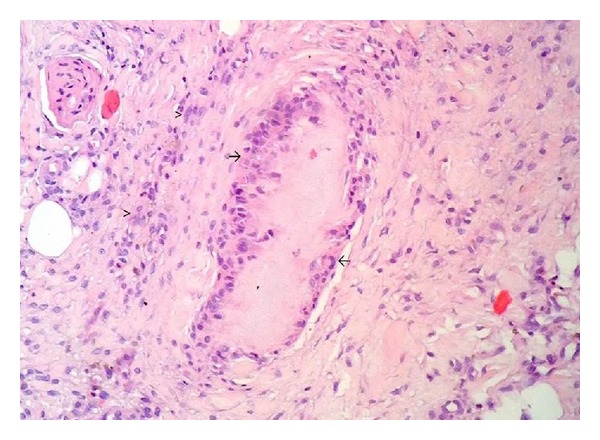
Histopathological findings of the subcutaneous nodule: foreign body cell reaction to eosinophilic fibrillar material consistent with gouty tophi (arrow). In the peripheral area, there is an inflammatory infiltrate of lymphocytes, plasma cells, and macrophages (arrowhead) (hematoxylin and eosin 100x).

The patient was a 41-year-old Brazilian mulatto male with a 10-year history of episodic arthritis and concomitant systemic arterial hypertension controlled with losartan 50 mg/daily. Arthritis initially affected the first right metatarsophalangeal joint followed by progressive involvement of other joints. During the past 5 years, he has experienced polyarthritis outbreaks affecting large and small joints associated with low-grade fever, but not morning stiffness. The most recent outbreak prompted his hospitalization at our institution. On admission, physical examination revealed a good general demeanor, low-grade fever of 37.8°C, and blood pressure of 130/80 mm/Hg. We did not identify abnormalities during the cardiorespiratory evaluation or hepatosplenomegaly on abdominal palpation. On musculoskeletal examination, we identified swan-neck deformity of the fingers that was reversible with passive movement (Figure 1). Additionally, ankles, knees, and elbows appeared swollen and felt warm to the touch. He had painless subcutaneous nodules of fibrous consistency and different sizes (0.5 to 1 cm) on the extensor surfaces of the forearms and legs. Laboratory tests results indicated hemoglobin of 8.5 g/dL, white blood cell count 12700 cells/mm^3^, and uricemia in the upper normal range (6.7 mg/dL), and he had normal glycemia, total cholesterol, creatinine, and hepatic enzymes. Acute phase reactants, including erythrocyte sedimentation rate and C-reactive protein, were 62 mm and 418 mg/L, respectively. Anti-CCP antibodies by ELISA were low-positive (38 U), with a negative cutoff value of <20 U. Antinuclear antibodies, rheumatoid factor, anti-dsDNA, and anti-SSA/Ro antibodies were negative. Plain radiographs of the hand were normal with no evidence of bone erosions (Figure 2). Biopsy of a subcutaneous nodule was performed and the histopathological findings were consistent with gouty tophi (Figure 3). The patient began treatment with prednisone 40 mg/daily and colchicine 1 mg/daily with progressive improvement of symptoms and normalization of acute phase reactants. During subsequent follow-ups at the outpatient clinic, he was tapered off prednisone and continued to receive maintenance treatment with colchicine; then, allopurinol was added to it. After approximately 1 year of follow-up, he is doing well, is receiving treatment with allopurinol only (300 mg/day), and has no complaints of joint pain or swelling.

## 3. Discussion

JA is characterized by a deforming arthropathy that can mimic the clinical presentation of rheumatoid arthritis (RA). The joint deformities most frequently observed in JA are ulnar deviation, swan neck, z-thumb, boutonniere, and hallux valgus, all similar to those observed in RA [1], except for their reversible quality. Generally, JA is not characterized by signs of erosion or bone destruction on plain radiograph, as observed in RA. However, in a minority of cases, the affected joints can present small erosions on magnetic resonance images [8].

Our patient presented the classical features of JA: reversible joint deformities, normal plain radiograph, and history of inflammatory arthritis. These corresponded with the recently proposed classification criteria for JA [9]. In JA, deformities are secondary to soft tissue abnormalities, including laxity of ligaments, fibrosis of the capsule, and muscular imbalance added to synovitis and tenosynovitis. Whether or not joint hypermobility can contribute to the development of JA is controversial. However, this clinical feature was not observed in our patient.

Gout has a typical joint arthritis distribution and clinical course (i.e., intermittent monoarthritis of large joints) and, probably, these are the reasons why JA is a rare occurrence in this disorder. Our patient had a unique and atypical presentation of gout, characterized by bouts of polyarthritis affecting small and large joints. Similarly, in RF, the most common pattern of joint involvement consists of migratory, self-limited arthritis affecting large joints. However, a few patients with RF have small joint involvement as well and may further develop JA.

It is important to emphasize that some cases of gout mimicking RA with involvement of peripheral joints or atlantoaxial subluxation have been reported. Furthermore, rare cases of coexisting gout and RA [10–13] have been described. However, the latter does not seem to be the case with our patient because he presented clinical deformities in the absence of significant radiological abnormalities. In his case, the presence of subcutaneous nodules could mislead the diagnosis and point towards RA, but the presence of uric acid crystals within the nodules excluded this hypothesis. Conversely, he had low-positive anti-CCP antibodies by ELISA with negative rheumatoid factor. Although the present clinical scenario is more compatible with the association of gout and JA as discussed above, based on the presence of anti-CCP antibodies, we cannot exclude the possibility of future RA development. This progression has occurred in a few cases of concomitant gout and RA, in which gout preceded the development of RA [14]. Alternatively, the presence of anti-CCP antibodies could be incidental despite the high specificity of this test for RA diagnosis; false positives can occur rarely in some clinical situations such as infectious disease [15].
